# Diseases of the Heart; Their Diagnosis and Treatment

**Published:** 1901-07

**Authors:** 


Diseases of the Heart; Their Diagnosis and Treatment. By Albert Abrams, A. M., M. D., San Francisco, Consulting Physician for Diseases of the Chest, Mt. Zion Hospital and the French Hospital. Illustrated. Pages, 172. Chicago: G. P. Engelhard & Co. 1900. [Price, $1.00.]

In this little volume, which treats the subject monographically, the author discusses the diseases of the heart from a practical aspect. Much stress is laid upon the careful painstaking examination necessary to arrive at a correct diagnosis and some of the author's most noteworthy researches in methods of diagnosis, are here recorded for the first time in collected form. The author takes up the treatment of these cases in a practical, methodical manner, going into the careful details, minor or secondary treatment.

The book is to be commended. The type and paper is excellent.

